# Multi-Omics Analysis Reveals the Hypolipidemic Effect of Dietary Fiber from *Auricularia heimuer* Residue

**DOI:** 10.3390/foods15213828

**Published:** 2026-10-26

**Authors:** Tianci Wang, Zikun Jia, Heriban Mei, Wenbo Yan, Ling Su, Qi Wang

**Affiliations:** 1 Engineering Research Center of Edible and Medicinal Fungi, Ministry of Education, Jilin Agricultural University, Changchun 130118, China; wangtianci0617@163.com (T.W.);; 2 College of Plant Protection, Jilin Agricultural University, Changchun 130118, China

**Keywords:** *Auricularia heimuer* residue, dietary fiber, hypolipidemic activity, intestinal flora, serum metabolomics

## Abstract

In this study, a structurally novel soluble dietary fiber (ASDF) was extracted from *Auricularia heimuer* residue. ASDF (340.747 kDa) is mainly composed of glucose and mannose, exhibiting a hollow globular morphology, semi-crystalline structure, and good thermal stability. In vitro, ASDF showed strong cholesterol adsorption and bile salt binding capacities (IC_50_ = 0.5731 and 0.5730 mg/mL), and effectively inhibited lipase, α-amylase, and α-glucosidase activities. In vivo, ASDF dose-dependently inhibited weight gain in HFD-induced obese mice and ameliorated dyslipidemia by decreasing TG, TC, AST, ALT, and LDL-C levels, while increasing HDL-C. It also alleviated hepatic steatosis by regulating the expression of *SREBP-1c*, *FAS* and *CPT-1*. Furthermore, 16S rRNA sequencing revealed that ASDF remodeled HFD-induced gut dysbiosis by enriching *Bacteroides* and *norank_f__Muribaculaceae*, and suppressing the overproliferation of *Akkermansia* and *Blautia*. Serum metabolomics indicated that ASDF primarily reversed abnormal metabolites by downregulating succinic acid and 20-hydroxyecdysone, and upregulating 13(S)-HODE, thereby regulating lipid metabolism, bile acid metabolism, and the PPAR signaling pathway. This study provides a theoretical basis for the high-value utilization of mushroom by-products and the development of lipid-lowering functional foods.

## 1. Introduction

Hyperlipidemia (HLP) is a systemic metabolic disorder characterized by abnormally elevated blood lipid levels [1]. With the improvement of living standards, the excessive intake of high-fat and high-cholesterol diets has led to a dramatic rise in the prevalence of obesity [2], which further exacerbates the development and progression of HLP. One of the most harmful effects of HLP is that it accelerates the progression of atherosclerosis [3]. Currently, HLP is a critical threat to human health. Epidemiological data indicate that the prevalence of dyslipidemia among Chinese adults has reached 35.6%, and globally, it contributes to millions of premature deaths annually [4]. Besides, the number of people suffering from diabetes mellitus, myocardial infarction, cerebral infarction, stroke and hemiplegia in China every year has been continuously increasing, mainly due to HLP [5]. Current therapeutic strategies for HLP primarily include enhancing reverse cholesterol transport and promoting the conversion of cholesterol to bile acids in the liver [6]. Medications used to treat HLP include statins, fibrates, bile acid sequestrants, and niacin [7]. However, these pharmacological treatments are associated with well-documented adverse effects, such as hepatotoxicity and nephrotoxicity, leading to poor patient tolerance [8]. Therefore, there is an urgent need to explore safe, natural dietary interventions with minimal side effects to prevent and ameliorate HLP.

Dietary fiber (DF) has emerged as an effective dietary intervention for HLP [9]. A large-scale meta-analysis of 181 randomized controlled trials involving 14,505 participants demonstrated that soluble fiber supplementation significantly reduced TC by 10.82 mg/dL and LDL-C by 8.28 mg/dL in adults [10]. Mechanistically, DF physically binds to bile acids and delays intestinal lipid absorption. Furthermore, because DF cannot be digested in the small intestine, it is fermented by the intestinal flora in the colon, enabling it to significantly modulate the microbial community [11]. This intestinal flora modulation is increasingly recognized as a core mechanism in the treatment of HLP [12]. Dysbiosis, characterized by an elevated *Firmicutes*/*Bacteroides* ratio and a decreased abundance of probiotics, exacerbates HLP through systemic low-grade inflammation, insufficient production of short-chain fatty acids, and abnormal bile acid metabolism [13]. This dysbiosis exacerbates lipid metabolism disorders through multiple pathways. Specifically, the abundance of pro-inflammatory bacteria such as *Blautia* increases, inducing a systemic low-grade inflammatory response and interfering with hepatic lipid synthase activity [14]. In addition, decreased abundance of butyric acid-producing bacteria leads to insufficient production of short-chain fatty acids, impairing their inhibitory effect on cholesterol synthesis [15]. Meanwhile, abnormal bile acid metabolism and imbalance in the proportion of secondary bile acids impair the activation of the FXR signaling pathway, affecting reverse cholesterol transport [16]. Further, fecal bacterial transplantation (FMT) resulted in significantly lower serum TC and LDL in a model of hyperlipidemia, and the mechanism involved a remodeling of bile acid metabolism by decreasing oxygenated bile acid levels as a result of elevated *Muribaculum* abundance [17]. Meanwhile, gavage of a synbiotic formulation can significantly improve serum lipid abnormalities and reduce body weight in HFD mice [18]. Similarly, oral gavage of *Enterococcus faecium* R0026 combined with *Bacillus subtilis* R0179 prevented obesity-associated hyperlipidemia and modulated intestinal flora in C57BL/6 mice [19]. However, extracting high-purity, highly active DF often requires complex processes and high costs, which limits its large-scale application.

*Auricularia heimuer* is a widely consumed edible and medicinal fungus. Current research on *A. heimuer* has primarily focused on its polysaccharides, which exhibit excellent antioxidant, immunomodulatory, and hypolipidemic activities [20]. However, the industrial extraction of polysaccharides generates large amounts of residue. This residue is typically discarded, causing resource waste and environmental pollution [21]. Notably, this underutilized residue still contains abundant bioactive dietary fibers. Melanin was obtained from *A. heimuer* dregs in nearly 12% yield with excellent antioxidant activity [22]. In addition, the soluble dietary fiber in *A. heimuer* residue was extracted by cellulase with a yield of 9.28% [23]. Polysaccharides of *A. heimuer* prepared enzymatically showed positive hypolipidemic activity [24]. In addition, the SDF of *A. auricula* complexed with phenol had hypolipidemic activity in vitro [25]. Therefore, extracting high-value SDF from *A. heimuer* residue not only provides a low-cost source of functional food ingredients but also offers a promising solution to the environmental issues associated with agricultural waste.

In summary, a structurally novel soluble dietary fiber was extracted from *A. heimuer* residue, and its structural characteristics and in vitro lipid-lowering activity were systematically evaluated. Subsequently, a high-fat diet-induced hyperlipidemic mouse model was established to investigate the in vivo hypolipidemic effects of ASDF. By integrating non-targeted metabolomics and 16S rRNA sequencing, the underlying mechanisms were further explored. This research not only realizes the high-value utilization of agricultural waste but also provides novel mechanistic insights into dietary fiber-mediated management of metabolic diseases.

## 2. Materials and Methods

### 2.1. Chemicals and Reagents

The experimental material was the residue remaining after hot water extraction of polysaccharides from *A. heimuer*. Cellulase (CAS 9012-54-8), Na_2_CO_3_ (CAS 497-19-8), cholesterol (CAS 57-88-5), 4-methylumbelliferyl oleate (CAS 18323-58-5), sodium glycocholate (CAS 863-57-0), and sodium taurocholate (CAS 145-42-6) were purchased from Beijing Chemical Reagent Factory (Beijing, China; analytical grade, AR). Potassium bromide (CAS 7758-02-3) was obtained from Tianjin Tianguang Co., Ltd. (Tianjin, China). α-Glucosidase (CAS 9001-42-7) was purchased from Shanghai Yuanye Bio-Technology Co., Ltd. (Shanghai, China). Pancreatic lipase (CAS 9001-62-1) was obtained from Shanghai Ruiyong Biotechnology Co., Ltd. (Shanghai, China). Pepsin (2500 U/mg, CAS 9001-75-6) and trypsin (CAS 9002-07-7) were purchased from Beijing Dingguo Changsheng Biotechnology Co., Ltd. (Beijing, China). α-Amylase (CAS 9000-90-2) was procured from Xingtai Wanda Biotechnology Co., Ltd. (Xingtai, China). Orlistat (CAS 96829-58-2) was obtained from Chongqing Zhien Pharmaceutical Co., Ltd. (Chongqing, China). Acarbose (CAS 56180-94-0) was purchased from Bayer HealthCare Pharmaceuticals (Leverkusen, Germany). The ALT (C009-2-1), AST (C010-2-1), LDL (A113-1-1), HDL (A112-1-1), TC (A111-1-1), TG (A110-1-1), and BCA (A045-4-2) kits were procured from Nanjing Jiancheng Bioengineering Institute (Nanjing, China; 96T, microplate method). All other reagents were of analytical grade.

### 2.2. The Extraction of Dietary Fibre from A. heimuer

The dietary fiber from *A. heimuer* residue was extracted by an enzymatic method with slight modifications [26]. Specifically, the *A. heimuer* residue was crushed and passed through an 80-mesh sieve. The dried fungal powder was accurately weighed and placed in a beaker, and distilled water was added at a ratio of 1:30. The pH was then adjusted to 5 with 0.05 mol/L HCl. Subsequently, 0.9% cellulase was added, and the entire reaction system was maintained at 50 °C for 3 h to allow enzymatic hydrolysis. After the reaction, the system was immediately placed in a 100 °C water bath for 5 min to inactivate cellulase. The supernatant was then separated by centrifugation at 6000 r/min for 15 min, and its pH was adjusted to 7 with 0.05 mol/L NaOH. Finally, anhydrous ethanol was added at four times the volume of the supernatant to induce precipitation, which was separated by centrifugation at 6000 r/min and collected as the dietary fiber. Total dietary fiber and soluble dietary fiber contents were determined by the enzymatic-gravimetric method according to GB 5009.88-2014 (National Food Safety Standard: Determination of Dietary Fiber in Foods) [27]. Crude polysaccharide content was measured by the phenol-sulfuric acid method. Protein content was determined by the Kjeldahl method according to GB 5009.5-2016 (National Food Safety Standard: Determination of Protein in Foods) [28]. The results showed that the total dietary fiber content was 59.60%, the soluble dietary fiber content was 59.10%, the crude polysaccharide content was 10.03%, and the total protein content was 19.10%. Since soluble dietary fiber accounted for the vast majority of the total dietary fiber (59.10%/59.60%, approximately 99.16%), it was identified as a soluble dietary fiber and named ASDF.

### 2.3. Structural Characterization of ASDF

#### 2.3.1. Molecular Weight and Monosaccharide Composition Analysis

The molecular weight of ASDF was determined by high-performance gel permeation chromatography coupled with evaporative light scattering detection (HPGPC-ELSD) (Agilent Technologies, Santa Clara, CA, USA) as previously described with slight modifications [29]. Briefly, ASDF was dissolved in 0.2 M NaCl at a concentration of 1 mg/mL and filtered through a 0.22 μm membrane. An aliquot of 20 μL was injected into a TSK-gel G-3000PWXL column (7.8 × 300 mm, Tosoh, Tokyo, Japan) maintained at 35 °C. The mobile phase was 0.2 M NaCl at a flow rate of 0.6 mL/min. The molecular weight was calculated using a calibration curve constructed with dextran standards of known molecular weights. The monosaccharide composition of ASDF was analyzed by ion exchange chromatography using a Dionex™ CarboPac™ PA20 column (4.6 × 250 mm, 5 μm; Thermo Fisher Scientific, Waltham, MA, USA) according to a previously reported method [30]. The column temperature was maintained at 25 °C, and the detection wavelength was 245 nm. The mobile phases consisted of (A) pure water, (B) 200 mM NaOH, and (C) 200 mM NaOH/500 mM NaAC. The flow rate was 0.5 mL/min, and the injection volume was 10 μL. The monosaccharide composition was identified and quantified by comparing retention times with standard monosaccharides.

#### 2.3.2. FT-IR and XRD Analysis

FT-IR spectra were recorded using a Nicolet FT-IR spectrometer (Thermo Fisher Scientific, Waltham, MA, USA). ASDF (1–2 mg) was mixed and ground with spectroscopic-grade potassium bromide (KBr) at a ratio of 1:100 (*w*/*w*) in an agate mortar, and then compressed into a translucent pellet under vacuum. A pure KBr pellet was used for background subtraction. Spectra were collected over the wavenumber range of 4000–400 cm^−1^ at a resolution of 4 cm^−1^, and 32 consecutive scans were co-added to enhance the signal-to-noise ratio [31]. In addition, XRD patterns of ASDF were obtained using a Rigaku SmartLab SE diffractometer (Rigaku, Tokyo, Japan) with a wavelength of 1.54056 Å. The diffraction angle (2θ) ranged from 5° to 90° at a scanning speed of 2°/min [32].

#### 2.3.3. TGA Analysis

TGA was performed on a Mettler TGA2 thermogravimetric analyzer (Mettler Toledo, Greifensee, Switzerland). Lyophilized ASDF (5–10 mg) was placed in an alumina crucible and heated from 30 to 600 °C at a heating rate of 10 °C/min under a continuous flow of dry nitrogen at 20 mL/min [33].

#### 2.3.4. SEM and AFM Analysis

The surface morphology of ASDF was observed using a SU8600 field emission scanning electron microscope (Hitachi, Tokyo, Japan). A small amount of dried ASDF powder was evenly dispersed on conductive tape, sputter-coated with gold for approximately 60 s, and loosely attached particles were blown off with an ear bulb. Images were captured at different magnifications [34]. The molecular morphology of ASDF was observed using a Bruker Dimension Icon atomic force microscope (Bruker, Karlsruhe, Germany). ASDF was dissolved in distilled water at a concentration of 10 μg/mL and filtered through a 0.45 μm membrane. An aliquot (20 μL) was dropped onto freshly cleaved mica and dried in an oven at 30 °C. Images were acquired in tapping mode at room temperature [35].

### 2.4. The Hypolipidemic Activity Assay of ASDF In Vitro

The lipid-lowering activity of ASDF was evaluated by determining its bile salt binding capacity, pancreatic lipase inhibitory activity, α-amylase inhibitory activity, and α-glucosidase inhibitory activity. The IC_50_ values for the in vitro assays were calculated by plotting the binding or inhibition rates against sample concentrations and performing nonlinear regression using a four-parameter logistic model in GraphPad Prism 9.

#### 2.4.1. Bile Salt Binding Capacity

The bile salt binding capacity of ASDF was determined according to the method described by Chen et al. [36] with slight modifications. Sodium glycocholate (SGC) and sodium taurocholate (STC) were separately dissolved to prepare 7 mmol/L solutions. A standard curve was constructed by mixing 2 mL of the SGC or STC solution with 6 mL of 60% H_2_SO_4_, heating the mixture at 70 °C for 20 min, cooling it, and measuring the absorbance at 387 nm. The resulting linear regression equations were y = 3.2815x + 0.6027 (R^2^ = 0.99405) for SGC and y = 2.7990x + 0.7305 (R^2^ = 0.99412) for STC. For the binding assay, 3 mL of ASDF solution was mixed with 3 mL of 10 mg/mL pepsin (pH 6.3) and 1 mL of 0.01 mol/L HCl, and the mixture was incubated at 37 °C for 1 h. The pH was then adjusted to 6.3, after which 4 mL of 10 mg/mL trypsin was added, and the mixture was incubated for another 1 h. Subsequently, 4 mL of 0.4 mmol/L SGC or 0.5 mmol/L STC was added to the mixture, which was then incubated at 37 °C for 1 h. The mixture was centrifuged at 4000 r/min for 20 min, and the residual bile salt in the supernatant was measured. The bile salt binding rate was calculated using the following equation: Binding rate (%) = (A_1_ − A_2_)/A_1_ × 100%, where A_1_ represents the amount of bile salt added and A_2_ represents the residual amount.

#### 2.4.2. Pancreatic Lipase Inhibitory Activity

The pancreatic lipase inhibitory activity was determined using 4-MUO as a substrate, following the method of Zhang et al. [37] with slight modifications, and orlistat was used as a positive control. Pancreatic lipase and 4-MUO were prepared at a concentration of 0.5 mg/mL in PBS. A 25 μL aliquot of ASDF solution and 25 μL of lipase solution were pre-incubated at 37 °C for 5 min. Then, 50 μL of 4-MUO was added to the mixture, which was allowed to react for 20 min. The reaction was terminated by adding 100 μL of 0.1 mol/mL sodium citrate. The fluorescence was measured at excitation and emission wavelengths of 320 nm and 450 nm, respectively. The inhibition rate was calculated using the following equation: Inhibition rate (%) = [1 − (A_1_ − A_2_)/(A_3_ − A_4_)] × 100%, where A_1_ is the absorbance of ASDF with active lipase, A_2_ is the absorbance of ASDF with inactivated lipase, A_3_ is the absorbance of PBS with active lipase, and A_4_ is the absorbance of PBS with inactivated lipase.

#### 2.4.3. α-Amylase Inhibitory Activity

The α-amylase inhibitory activity was determined by the DNS method according to the procedure described by Li et al. [38] with slight modifications, using acarbose as a positive control. A 0.2 mL aliquot of ASDF solution was mixed with 0.4 mL of α-amylase and incubated at 37 °C for 10 min. Then, 0.5 mL of 5% starch solution was added and allowed to react for 10 min. Subsequently, 2 mL of DNS reagent was added to the mixture, which was heated at 100 °C for 15 min. After cooling, the absorbance was measured at 540 nm, and the value was recorded as A_1_. The absorbance of the acarbose group was recorded as A_2_, and the absorbance of the blank group was recorded as A_0_. The inhibition rate was calculated using the following equation: Inhibition rate (%) = [1 − (A_1_ − A_2_)]/A_0_ × 100%.

#### 2.4.4. α-Glucosidase Inhibitory Activity

The α-glucosidase inhibitory activity was determined according to the method of Ren et al. [39] with slight modifications, with acarbose used as a positive control. A 50 μL aliquot of ASDF solution was mixed with 100 μL of α-glucosidase (0.5 U/mL) and incubated at 37 °C for 10 min. Then, 100 μL of 5 mmol/L PNPG was added and allowed to react for 20 min. The reaction was terminated by adding 1 mol/L Na_2_CO_3_, and the absorbance was measured at 405 nm, which was recorded as A_1_. The absorbance of the mixture containing PNPG and ASDF without enzyme was recorded as A_2_, and the absorbance of the mixture containing PNPG and enzyme without sample was recorded as A_3_. The inhibition rate was calculated using the following equation: Inhibition rate (%) = [1 − (A_1_ − A_2_)]/A_3_ × 100%.

### 2.5. The Animal Model Establishment and Treatment

All experimental procedures were approved by the Laboratory Animal Ethics Committee of Jilin Agricultural University (ethical approval code: 20240613001), and the animal experiments were conducted in strict accordance with the ARRIVE guidelines. Sixty healthy male Kunming mice (6–8 weeks old) were acclimatized for 7 d under controlled conditions (temperature 20–25 °C, humidity 50–55%, and a 12 h light/dark cycle) with free access to food and water. After acclimatization, 10 mice were randomly selected as the normal control group (NC) and continued to receive the basal diet. The remaining mice were fed a high-fat diet (D12492, 60 kcal% fat; Research Diets, Inc., New Brunswick, NJ, USA) to induce obesity. After 8 weeks of high-fat feeding, the obesity degree was calculated as: obesity degree (%) = (body weight of the experimental group—average body weight of the NC group)/average body weight of the NC group × 100%. Mice with an obesity degree > 20% were considered successfully modeled, which is consistent with the widely accepted criterion for diet-induced obesity in rodent models [40]. All high-fat-fed mice met this criterion and were included in the subsequent experiment.

The obese mice were randomly divided into five groups (*n* = 10 per group): the model control group (MC), positive control group (PC), ASDF low-dose group (ASDFL), medium-dose group (ASDFM), and high-dose group (ASDFH). The NC group continued on the basal diet, while the experimental groups remained on the high-fat diet. The PC group was gavaged with orlistat (150 mg/kg), and the ASDFL, ASDFM, and ASDFH groups were gavaged with ASDF at 100, 200, and 400 mg/kg, respectively. The intervention period lasted for 6 weeks. At the end of the experiment, the mice were fasted for 12 h with free access to water. Blood samples were collected via retro-orbital puncture, after which the mice were euthanized by cervical dislocation, in accordance with the AVMA Guidelines for the Euthanasia of Animals: 2020 Edition. The blood samples were centrifuged at 4 °C and 2000 r/min for 10 min, and the serum was collected and stored at −80 °C. The liver tissues were fixed in 4% paraformaldehyde, and the remaining tissues and feces were stored at −80 °C for further analysis.

### 2.6. The Histological Analyses

Liver tissues were fixed in 4% paraformaldehyde at room temperature for 24 h. After being washed with PBS, the tissues were dehydrated through a graded ethanol series, cleared with xylene, and embedded in paraffin. The paraffin blocks were sectioned into 4 μm thick slices using a microtome (RM2235, Leica Biosystems, Wetzlar, Germany). The sections were deparaffinized, rehydrated, and stained with hematoxylin and eosin (H&E) [41]. After dehydration, clearing, and coverslipping, the stained sections were observed under a light microscope (Eclipse CI, Nikon, Tokyo, Japan) at a total magnification of 400× (40× objective lens). Images were captured using a digital imaging system (DS-U3, Nikon, Tokyo, Japan). ImageJ software (version 1.53t, National Institutes of Health, Bethesda, MD, USA) was used to add scale bars to the images.

### 2.7. The Biochemical Indicators Analysis

Liver tissues were mixed with 0.1 M PBS solution at a volume ratio of 1:9 [42]. The samples were thoroughly ground using a high-throughput tissue grinder (Tissuelyser-24, Shanghai Jingxin, Shanghai, China) at 4 °C and 60 Hz for 45 s, followed by centrifugation at 4 °C and 3000 r/min for 15 min to collect the supernatant. The levels of serum TC, TG, HDL, LDL, AST, and ALT were measured using commercial assay kits, strictly following the manufacturer’s protocols. All measurements were conducted using the 96-well microplate method based on standard colorimetric/enzymatic reactions. The absorbance was read using a microplate reader (SpectraMax iD3, Molecular Devices, San Jose, CA, USA), and the concentrations were calculated according to the respective standard curves.

### 2.8. Real-Time Quantitative PCR

Total RNA was extracted from the liver tissues using a commercial RNA extraction kit (Servicebio, Wuhan, China). The RNA concentration and purity were determined using a NanoDrop 2000 spectrophotometer (Thermo Fisher Scientific, Waltham, MA, USA). For cDNA synthesis, 1 μg of total RNA was reverse-transcribed using the Servicebio RT First Strand cDNA Synthesis Kit (G3330) in a 20 μL reaction system. The reaction was performed at 25 °C for 5 min, 42 °C for 30 min, and 85 °C for 5 s.

Quantitative PCR was conducted using 2× SYBR Green qPCR Master Mix (Servicebio, G3320) on a Bio-Rad CFX Real-Time PCR System. The 15 μL reaction mixture contained 7.5 μL of 2× qPCR Mix, 1.5 μL of specific primers, 2.0 μL of cDNA, and 4.0 μL of Nuclease-Free Water. The amplification protocol consisted of an initial denaturation at 95 °C for 10 min, followed by 40 cycles of 95 °C for 15 s and 60 °C for 30 s. A melting curve analysis (65 °C to 95 °C) was performed to verify amplification specificity. The relative mRNA expression levels of target genes (*FAS*, *SREBP-1c*, and *CPT-1*) were calculated using the 2^−ΔΔCt^ method and normalized to the internal control gene. The primer sequences are listed in Table 1.

### 2.9. Analysis of the Intestinal Flora

Total genomic DNA was extracted from the cecum contents using the TGuide S96 Stool DNA Kit (Tiangen Biotech, Beijing, China) according to the manufacturer’s instructions. The concentration and purity of the extracted DNA were determined using a NanoDrop 2000 UV-Vis spectrophotometer (Thermo Scientific, Wilmington, DE, USA). The hypervariable V3–V4 region of the bacterial 16S rRNA gene was amplified using the primer pair 338F (5′-ACTCCTACGGGAGGCAGCA-3′) and 806R (5′-GGACTACHVGGGTWTCTAAT-3′). The PCR reaction was performed in a 20 μL system containing 5–50 ng of DNA template, 0.3 μL of each primer (10 μM), 5 μL of KOD FX Neo Buffer, 2 μL of dNTPs (2 mM), and 0.2 μL of KOD FX Neo polymerase. The amplification conditions were: initial denaturation at 95 °C for 5 min; 20 cycles of 95 °C for 30 s, 50 °C for 30 s, and 72 °C for 40 s; and a final extension at 72 °C for 7 min. The PCR products were purified using the Omega DNA purification kit (Omega Inc., Norcross, GA, USA) and quantified with a Qsep-400 (BiOptic, New Taipei City, Taiwan, China). The amplicon library was paired-end sequenced (2 × 250 bp) on an Illumina MiSeq platform (Biomarker Technologies Co., Ltd., Beijing, China). For bioinformatics analysis, the raw data were processed using the BMKCloud platform (https://www.biocloud.net). Raw reads were primarily filtered using Trimmomatic (version 0.33) based on nucleotide quality. Primer sequences were identified and removed using Cutadapt (version 1.9.1). The paired-end reads were assembled using USEARCH (version 10.0), followed by chimera removal using UCHIME (version 8.1). The high-quality sequences with >97% similarity were clustered into operational taxonomic units (OTUs) using USEARCH. Taxonomy annotation of the OTUs was performed based on the Naive Bayes classifier in QIIME2 using the SILVA database (release 138.1) with a confidence threshold of 70%. Alpha diversity indices, including the Shannon, Simpson, ACE, and Chao indices, were calculated using QIIME2 software to evaluate the complexity of species diversity within each sample. Beta diversity was analyzed by principal coordinate analysis (PCoA) to assess the structural differences in microbial communities among samples. One-way analysis of variance (ANOVA) was used to compare bacterial abundance and diversity. Linear discriminant analysis (LDA) coupled with effect size (LEfSe) was applied to evaluate the differentially abundant taxa, with a logarithmic LDA score of 4.0 set as the threshold.

### 2.10. The Analysis of the Non-Target Metabolome

The metabolomics analysis was performed using a Waters Acquity I-Class PLUS Ultra-High Performance Liquid Chromatography system coupled with a Waters Xevo G2-XS QTOF high-resolution mass spectrometer (Waters Corporation, Milford, MA, USA). Separation was achieved on an Acquity UPLC HSS T3 column (1.8 μm, 2.1 × 100 mm, Waters Corporation, Milford, MA, USA) at a flow rate of 400 μL/min. The mobile phase consisted of 0.1% formic acid in water (A) and 0.1% formic acid in acetonitrile (B). The gradient elution program was as follows: 0–0.5 min, 5% B; 5.5 min, 50% B; 9.0 min, 95% B; 10.5 min, 95% B; and 12.0 min, 5% B. The injection volume was 2 μL. Mass spectrometry data were acquired in MSe mode under the control of MassLynx V4.2 software, utilizing both low and high collision energy channels. The mass-to-charge ratio (*m*/*z*) scanning range was set to 50–1200, and the high collision energy was ramped from 10 to 40 V with a scanning frequency of 0.2 s per spectrum. The electrospray ionization (ESI) source was operated in both positive and negative ion modes. The key ESI parameters were as follows: capillary voltage, 2500 V (positive) or −2000 V (negative); cone voltage, 30 V; ion source temperature, 100 °C; desolvation gas temperature, 500 °C; backflush gas flow, 50 L/h; and desolvation gas flow, 800 L/h. The raw data were processed using Progenesis QI software (version 3.0) (Waters) for peak extraction, peak alignment, and compound identification based on the online METLIN database, public databases, and an in-house database. After normalizing the peak area to the total peak area, principal component analysis (PCA) and Spearman correlation analysis were used to evaluate the stability and repeatability of the samples and QC samples. The identified compounds were annotated for classification and pathway information using the KEGG, HMDB, and LipidMaps databases. Orthogonal partial least squares-discriminant analysis (OPLS-DA) was performed using the “ropls” package in R, and the reliability of the model was verified by 200 permutation tests. Differential metabolites were screened based on the criteria of VIP > 1, FC > 1 (or FC < 1), and *p* < 0.05. The significant KEGG pathway enrichments were calculated using the hypergeometric distribution test. Finally, a Spearman correlation analysis was performed to investigate the relationships between the differential metabolites and the intestinal flora.

### 2.11. Statistics Analysis

All experiments were conducted at least three times independently. Data are presented as the mean ± standard error of the mean (SEM). For comparisons among multiple groups, a one-way analysis of variance (ANOVA) followed by Duncan’s multiple range test was used, with a significance threshold set at *p* < 0.05. For association analysis, Spearman’s rank correlation coefficient was calculated. Statistical analyses and data visualization were performed using IBM SPSS Statistics 26 (IBM Corp., Armonk, NY, USA), GraphPad Prism 9 (GraphPad Software, San Diego, CA, USA), and the online BMKCloud platform (https://www.biocloud.net). Specifically, IBM SPSS Statistics 26 was used to perform the one-way ANOVA and Duncan’s post hoc tests for the physiological and biochemical datasets, including mouse body weight, organ indices, and serum parameters (TC, TG, HDL, LDL, AST, and ALT). GraphPad Prism 9 was utilized for generating bar graphs of these physiological datasets, as well as for conducting the nonlinear regression to calculate IC_50_ values and plot the dose–response curves for the in vitro bile salt binding and enzyme inhibition assays. The online BMKCloud platform was employed for the bioinformatics and multivariate statistical analyses of the 16S rRNA sequencing and metabolomics data. The datasets processed via this platform included the generation of PCA score plots, OPLS-DA score plots, volcano plots, KEGG pathway enrichment bubble plots, LEfSe cladograms, LDA bar charts, and Spearman correlation heatmaps.

## 3. Results and Discussion

### 3.1. Molecular Weight and Monosaccharide Composition of ASDF

**Figure 1:**
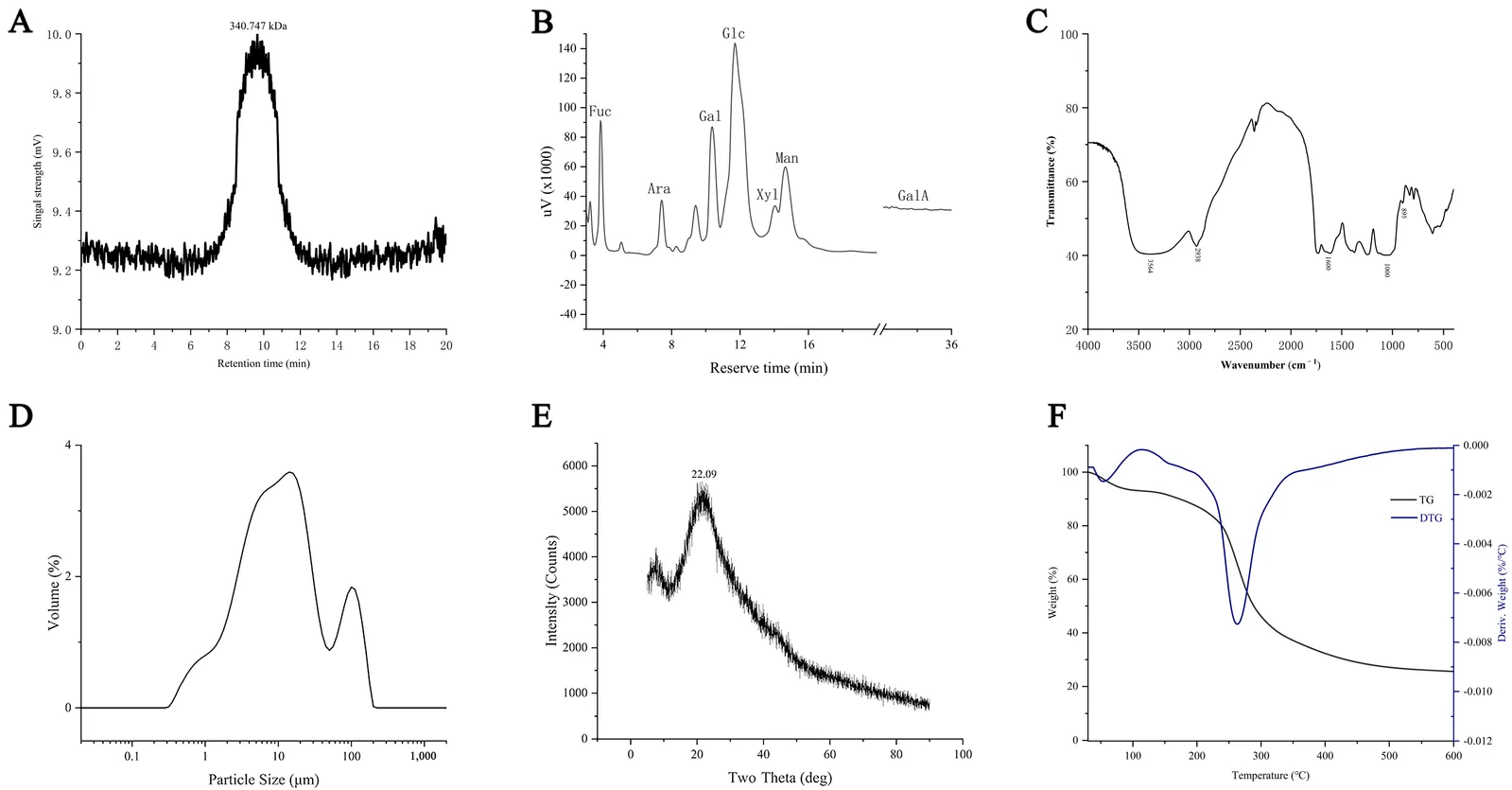
(**A**) Molecular weight, (**B**) monosaccharide composition, (**C**) FT-IR spectrum, (**D**) particle size, (**E**) XRD spectrum and (**F**) TGA spectrum of ASDF.

Molecular weight (Mw) is a fundamental parameter that profoundly affects the physicochemical properties and biological efficacy of soluble dietary fiber [43]. As shown in the HPGPC chromatogram (Figure 1A), ASDF exhibited a single, symmetrical elution peak, indicating that its component distribution is quite homogeneous. The retention time was recorded at 9.652 min, which was substituted into the standard regression equation lgMw = −0.2409x + 7.8576, R^2^ = 0.9947, yielding an Mw of 340.747 kDa. The structural function of dietary fiber is highly dependent on its molecular size. Excessively high Mw may cause steric hindrance and restricted diffusion, while excessively low Mw may fail to establish sufficient binding sites [44]. Dietary fibers with molecular weights in the range of 100–500 kDa generally exhibit superior lipid adsorption and bile salt binding capacities [45].

Monosaccharide composition is also a key factor determining the functional properties of soluble dietary fiber. As shown in Figure 1B, ASDF is a heteropolysaccharide, primarily composed of glucose (Glu, 60.92%), followed by mannose (Man, 18.10%), galactose (Gal, 8.14%), arabinose (Ara, 3.10%), xylose (Xyl, 1.83%), and galacturonic acid (GalA, 0.05%). Dietary fibers rich in glucose and mannose possess significant lipid-lowering activities [46]. In addition, GalA provides free carboxyl groups. It has been reported that higher uronic acid content is positively correlated with enhanced bile salt binding activity, because carboxyl groups can dissociate under intestinal pH conditions and enhance binding through electrostatic interactions and hydrogen bonds [45]. Therefore, ASDF may have good lipid-lowering potential.

### 3.2. Infrared Analysis and Particle Size of ASDF

Figure 1C showed that ASDF possessed the characteristic absorption peaks of cellulose. The broad absorption peak near 3564 cm^−1^ was generated by the stretching vibration of the O-H bond, and the peak near 2938 cm^−1^ was caused by the C-H resonance of the methyl group in the sugar ring. The characteristic peaks near 1600 cm^−1^ for carboxyl or aldehyde groups indicated the presence of glyoxylates in the residues, which is consistent with the detection of GalA. Additionally, the stretching vibration caused by the C-O-C of the pyranose ring was observed near 1000 cm^−1^, and the C-H angular vibration at 895 cm^−1^ was attributed to the β-glycosidic bond [47]. The prevalence of β-glycosidic bonds is structurally significant; compared with α-glycosidic bonds, they typically induce a more extended and rigid chain conformation [48], which may provide a stable spatial framework for entrapping lipid molecules. Particle size is a key factor affecting the structural and physicochemical properties of dietary fibers [49], which further modulates the accessibility of these chemical binding sites. As depicted in Figure 1D, the curves showed a significantly single-peaked distribution in the 1–100 μm interval, indicating that the main body of ASDF particles was concentrated in this range. The D(3,2) and D(4,3) of ASDF were 4.254 μm and 23.216 μm, respectively. Notably, ASDF had a smaller particle size compared to oat bran dietary fiber (35.39 μm), which showed significant lipid-lowering activity [50]. Structurally, the moderate molecular weight provides the necessary chain length, while the relatively small particle size prevents these macromolecular chains from tightly aggregating into dense, inaccessible structures [51]. Instead, they form a looser and more expanded matrix [52]. As the particle size decreases, the hydrophilic groups remain more exposed, which not only facilitates water binding [53], but also results in more loosely arranged fiber structures, increasing the surface area available for oil absorption and the ability to bind to glucose [54]. The synergistic effect of moderate Mw and small particle size substantially increases the specific surface area of ASDF. Consequently, more hydrophilic groups and physical adsorption sites are exposed, which is mechanistically crucial for capturing cholesterol, bile salts, and lipases.

### 3.3. X-Ray and TGA Analysis of ASDF

X-ray can reflect the crystal structure of dietary fibers through different diffraction phenomena, where sharp characteristic diffraction peaks reflect ordered crystalline regions and diffuse diffraction reflects disordered amorphous regions [55]. ASDF showed diffuse diffraction over most of the diffraction angle 2θ in the range of 0–90° (Figure 1E), and exhibited a strong diffraction peak at 2θ of 22.09°, which is consistent with the typical cellulose type I crystal structure widely reported for dietary fibers [56]. However, it should be noted that NMR analysis was not performed in this study to further confirm the anomeric configuration and glycosidic linkages, which will be addressed in our future work. Based on the XRD pattern, ASDF was judged to be in a semi-crystalline state.

TGA was used to evaluate the thermal degradation behavior of ASDF (Figure 1F). According to the TG and DTG curves, ASDF showed three weight loss stages in the range of 30–600 °C. The initial weight loss stage was from 30 to 112 °C, with a mass loss of 6.98%, which was attributed to water loss and the evaporation of some volatile organic components [57]. This stage was supported by a DTG peak temperature at 54.09 °C. The second weight loss stage was 120–400 °C, with a significant weight loss of 60.78%. The DTG curve showed a prominent peak at 262.20 °C, indicating that high temperatures induced the rapid degradation of polysaccharides and lipids, as well as strong reactions such as dehydroxylation, deoxygenation, or decarboxylation [58]. In the third stage (400–600 °C), a mass loss of 6.72% occurred, which was attributed to the degradation of cellulose or the polymerization products from the previous steps [59]. At this stage, the DTG curve became relatively flat, indicating a slower and more stable degradation rate.

### 3.4. SEM Scanning and AFM Scanning Analysis of ASDF

**Figure 2:**
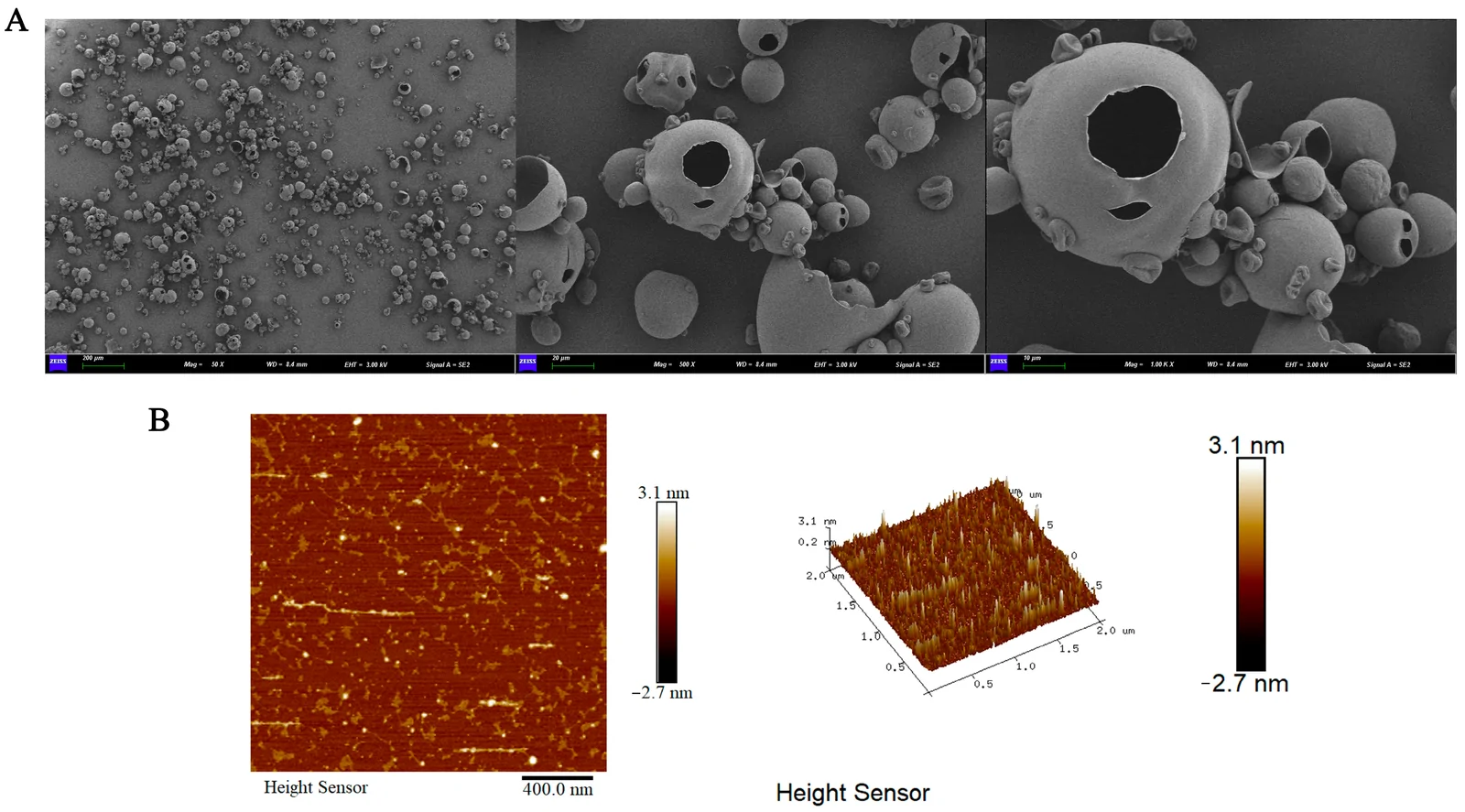
(**A**) SEM scanning and (**B**) AFM scanning of ASDF.

The surface morphology of ASDF was observed using scanning electron microscopy (SEM). As shown in Figure 2A, ASDF exhibited a hollow spherical structure with a porous surface. This morphology contrasts with the looser, porous lamellar structure of previously reported *A. heimuer* dietary fiber [60]. This difference is likely attributable to the cellulase-assisted extraction process, which disrupted the glycosidic bonds and intermolecular oxygen bonds within the polysaccharide chains [61], thereby breaking down the compact structure and giving rise to this unique hollow spherical shape. Furthermore, ASDF had an average particle size of 23.216 μm with a narrow distribution (Figure 1D). Although the particle size is in the micron range, the combination of this uniform size with the hollow and porous architecture is expected to provide a larger effective contact area and more binding sites compared to solid particles of the same diameter [62]. An increase in porosity and surface area enhancing adsorption capacity has also been observed in modified dietary fibers [63]. Meanwhile, the exposure of polar groups and the increase in surface area contribute to the physical entrapment of cholesterol and bile salts [64]. Therefore, this structural feature may contribute to the hydration, swelling, and adsorption properties [65] of ASDF, providing a structural basis for its hypolipidemic activity.

To further elucidate the molecular morphology and aggregation behavior, AFM was employed [66]. As shown in Figure 2B, ASDF molecules appeared as long, straight chains with multiple flexible branches, forming a locally aggregated, disordered network in aqueous solution, with a molecular height of approximately 3.1 nm. This aggregation is primarily driven by the abundant hydroxyl groups in ASDF, which facilitate strong intra- and intermolecular hydrogen bonds [67]. Notably, AFM observations revealed that ASDF preferred a loose and continuous spherical conformation rather than a highly compact one, which is in good agreement with the SEM results. Furthermore, the hindered formation of intermolecular hydrogen-bonding networks between crystalline particles and amorphous regions [68] further corroborates the semi-crystalline nature of ASDF, which is consistent with the XRD analysis. Collectively, the SEM and AFM results demonstrate that ASDF possesses a unique porous hollow spherical morphology and a semi-crystalline, highly branched structure, which may collectively contribute to its functional and hypolipidemic properties.

### 3.5. Hypolipidemic Activity Analysis of ASDF In Vitro

**Figure 3:**
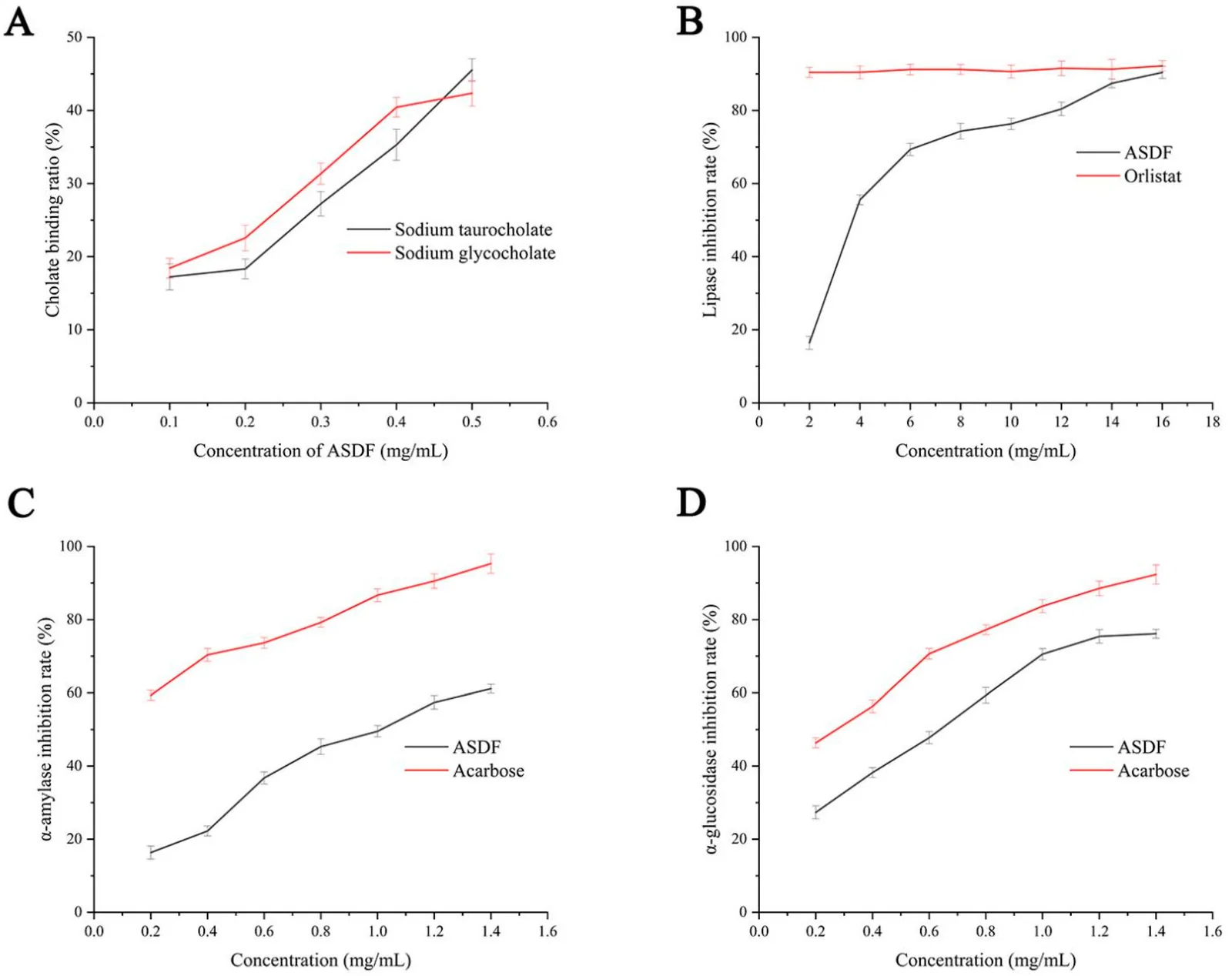
Hypolipidemic activity of ASDF in vitro: (**A**) cholate binding capacity, (**B**) lipase inhibition, (**C**) α-amylase inhibition and (**D**) α-glucosidase inhibition.

Bile acid sequestration is a primary mechanism by which dietary fibers lower serum cholesterol. By binding bile acids in the intestine, dietary fibers promote their fecal excretion, which forces the liver to consume endogenous cholesterol to synthesize new bile acids, ultimately lowering serum cholesterol levels [69]. As shown in Figure 3A, the binding rates of ASDF to sodium glycocholate and sodium taurocholate increased in a concentration-dependent manner. The calculated IC_50_ values were 0.5731 mg/mL and 0.5730 mg/mL, respectively, indicating a stronger binding capacity than that of lotus root polysaccharides [70]. Compared with lotus root polysaccharide (246 kDa), the larger molecular weight of ASDF (340.747 kDa) provides a more extended spatial network and abundant hydroxyl groups for binding interactions. Furthermore, the unique hollow spherical morphology with a porous surface (Figure 2A) offers additional physical space for the entrapment of bile acid molecules.

Pancreatic lipase is the key enzyme responsible for hydrolyzing dietary triglycerides into absorbable fatty acids [71]. Inhibiting this enzyme can effectively reduce intestinal lipid absorption [72]. As shown in Figure 3B, ASDF exhibited a strong inhibitory effect on pancreatic lipase, reaching 90.12% at 16 mg/mL, which was comparable to the positive control, orlistat (88.84%). The IC_50_ value was 3.2581 mg/mL, significantly lower than that of *Physalis alkekengi* L. calyx soluble dietary fiber (16.95 mg/mL) [73]. This strong inhibition is likely attributed to the encapsulation of the enzyme or its substrates within the highly branched and porous network of ASDF, thereby restricting their interaction and delaying lipolysis. Delaying carbohydrate digestion by inhibiting α-amylase and α-glucosidase is an effective strategy for managing postprandial blood glucose [74]. As illustrated in Figure 3C,D, ASDF exhibited dose-dependent inhibition of both enzymes, although the effects were weaker than those of acarbose. The IC_50_ values for α-amylase and α-glucosidase inhibition were 1.0345 mg/mL and 0.5901 mg/mL, respectively. Dietary fibers can inhibit these enzymes by physically wrapping the enzymes and substrates within the fiber network [75,76]. The increased surface area and porous structure of ASDF likely facilitate the embedding of starch and enzymes within its matrix, thereby retarding enzymatic activity [76]. The stronger inhibitory effects of ASDF compared to oat bran dietary fiber may be closely related to its unique spherical morphology [50]. Overall, ASDF demonstrates favorable in vitro hypolipidemic activity.

### 3.6. Effect of ASDF on HFD-Fed Mice

**Figure 4:**
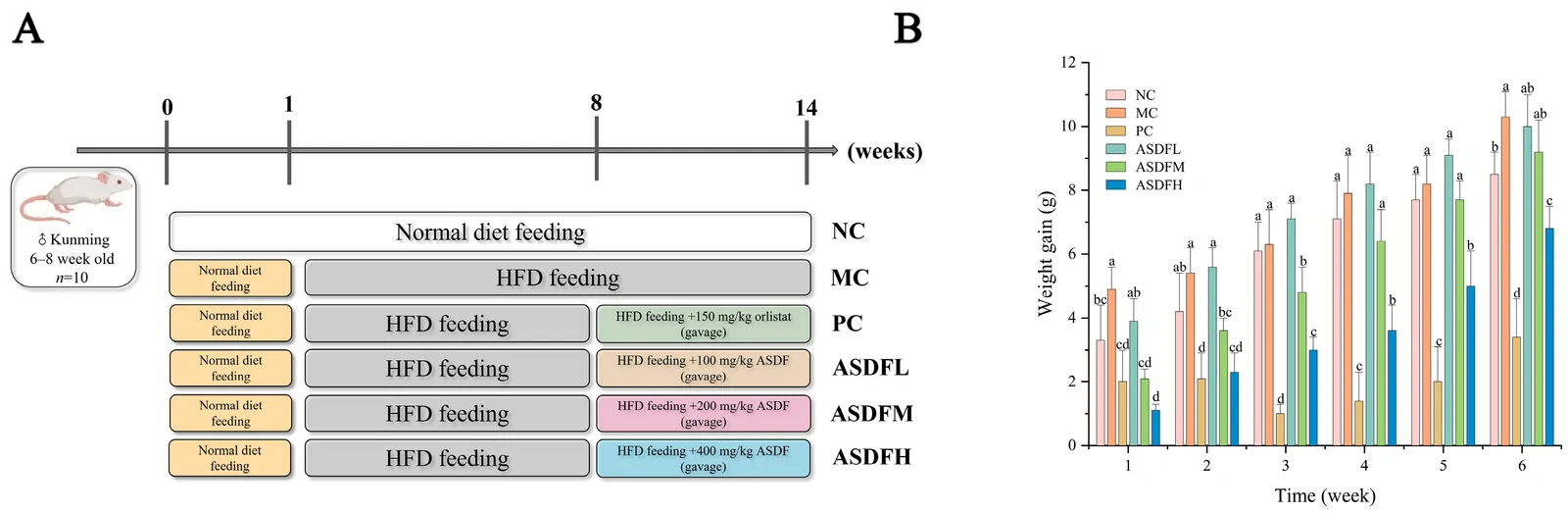
(**A**) Animal experimental protocols and (**B**) weight gain graph. Means with different letters indicate significant differences (*p* < 0.05).

From the initiation of ASDF intervention to the end of the experiment, the weight gain of mice was recorded weekly (Figure 4B). After high-fat feeding, the weight gain in the MC group was significantly higher than that in the NC group. Compared with the MC group, the ASDF-intervened and PC groups exhibited a slower trend of weight gain. By the final week, the weight gain in all ASDF-treated groups was significantly lower than that in the MC group, among which the ASDFH group showed the most prominent inhibitory effect, being secondary only to the PC group.

Furthermore, compared to the NC group, the organ indices (especially the liver index) and adiposity indices (inguinal and epididymal fat) in the MC group were significantly increased (Table 2), and the adiposity level nearly doubled due to severe fat accumulation. Interventions with different concentrations of ASDF and orlistat significantly alleviated these burdens. Notably, with the exception of the spleen index, the organ and adiposity indices in the ASDFH group were lower than those in the ASDFM group, and significantly lower than those in the ASDFL group. This indicates that ASDF can control weight gain and alleviate hepatic and adipose burdens in obese mice in a dose-dependent manner, which is similar to the effect of high-performance inulin dietary fiber [77].

### 3.7. Effect of ASDF on Blood Lipid Levels in HFD-Fed Mice

**Figure 5:**
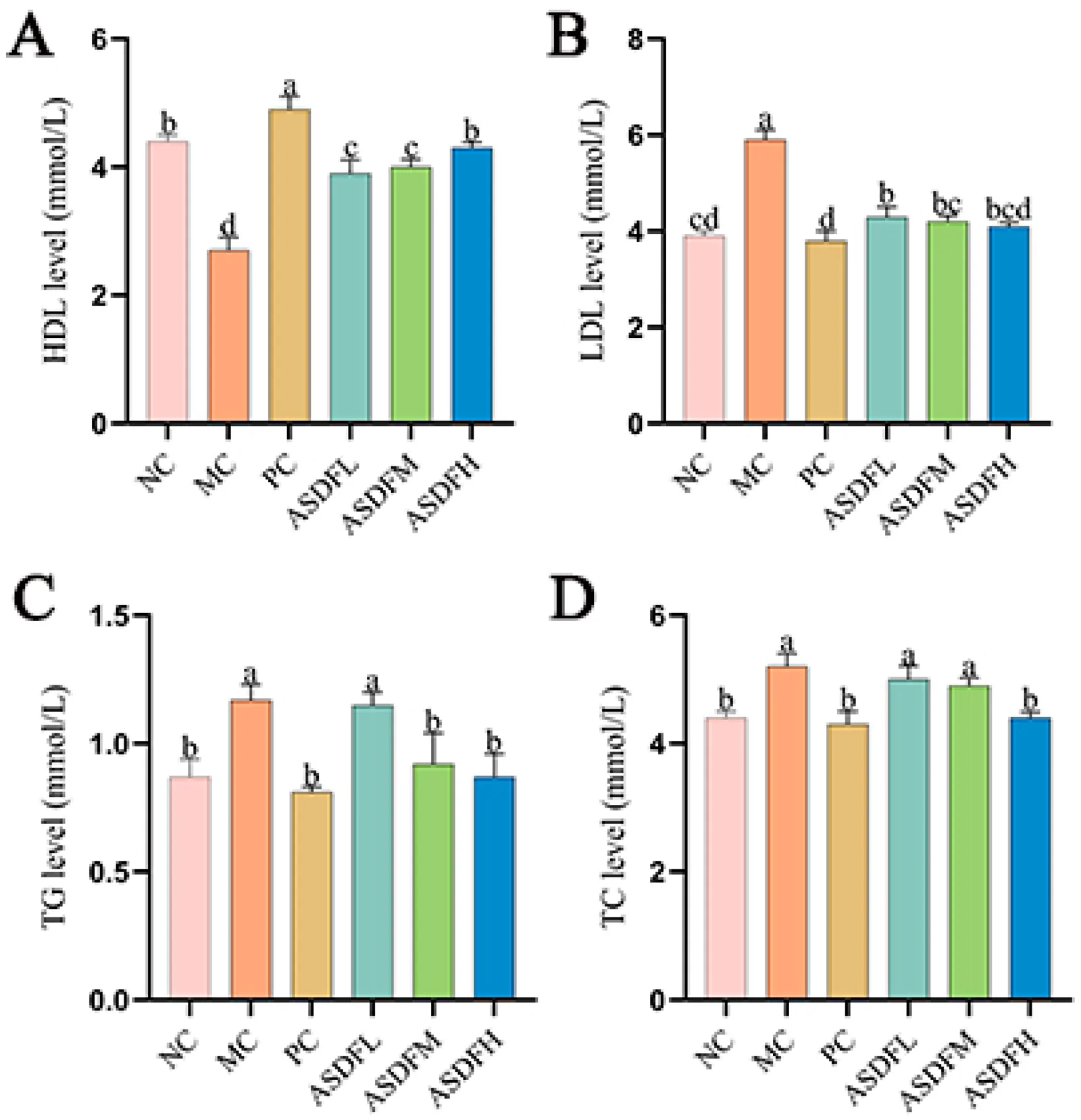
Effects of ASDF on lipid levels in mice: (**A**) HDL level, (**B**) LDL level, (**C**) TG level and (**D**) TC level. Means with different letters indicate significant differences (*p* < 0.05).

Hyperlipidemia is a disorder of abnormal lipid metabolism, and serum TC and TG levels are key indicators for monitoring lipids [78]. Elevated LDL and TG, or reduced HDL levels, are strongly associated with a high risk of cardiovascular disease [79]. Compared with the NC group, the MC group exhibited a severe lipid metabolism disorder. Specifically, serum TC, TG, and LDL levels in the MC group were elevated by approximately 1.18-fold, 1.34-fold, and 1.51-fold, respectively, while HDL levels were decreased by 38.6% (Figure 5). From a clinical and biological perspective, these magnitudes of change carry substantial prognostic significance. A 1-SD increase in genetically predicted TG levels was associated with a 60% increased risk of coronary artery disease [80]. Besides, each 1 mmol/L increase in LDL-C is associated with an approximately 20–30% increased risk of coronary heart disease events [81]. Individuals with low HDL-C had a 28% higher risk of incident myocardial infarction compared with those with normal HDL-C [82]. This combined lipid profile, elevated LDL-C and TG with drastically reduced protective HDL, represents a state of systemic lipid overload that drives the initiation and progression of atherosclerosis and elevates cardiovascular risk [83]. ASDF intervention generally improved these parameters, with ASDFH showing the most favorable numerical outcomes, but statistical significance varied among indices. For LDL and HDL, all ASDF-treated groups showed significant improvements compared with the MC group. However, no significant differences were observed between ASDFM and ASDFH, indicating that a moderate dose of ASDF was sufficient to elicit a near-maximal regulatory effect on these lipoproteins. For TC and TG, more pronounced threshold effects were observed. For TC, although ASDFL and ASDFM showed a downward numerical trend, neither reached statistical significance compared with the MC group. Only ASDFH significantly reduced TC levels. For TG, while ASDFL showed a non-significant decreasing trend, both ASDFM and ASDFH achieved significant reductions compared with the MC group, with no significant difference between them. This phenomenon suggests that achieving systemic reductions in TC and TG requires a relatively high intestinal concentration of ASDF to provide sufficient physical entrapment of lipids and bile salts, as supported by our in vitro adsorption assays. Similarly to high-performance *Hericium erinaceus* dietary fiber [84], the dose-dependent and threshold-based regulation of lipid metabolic pathways is a key characteristic of ASDF’s lipid-lowering effects.

### 3.8. Effect of ASDF on Liver Function, Pathology, and Lipid Metabolism-Related Gene Expression

**Figure 6:**
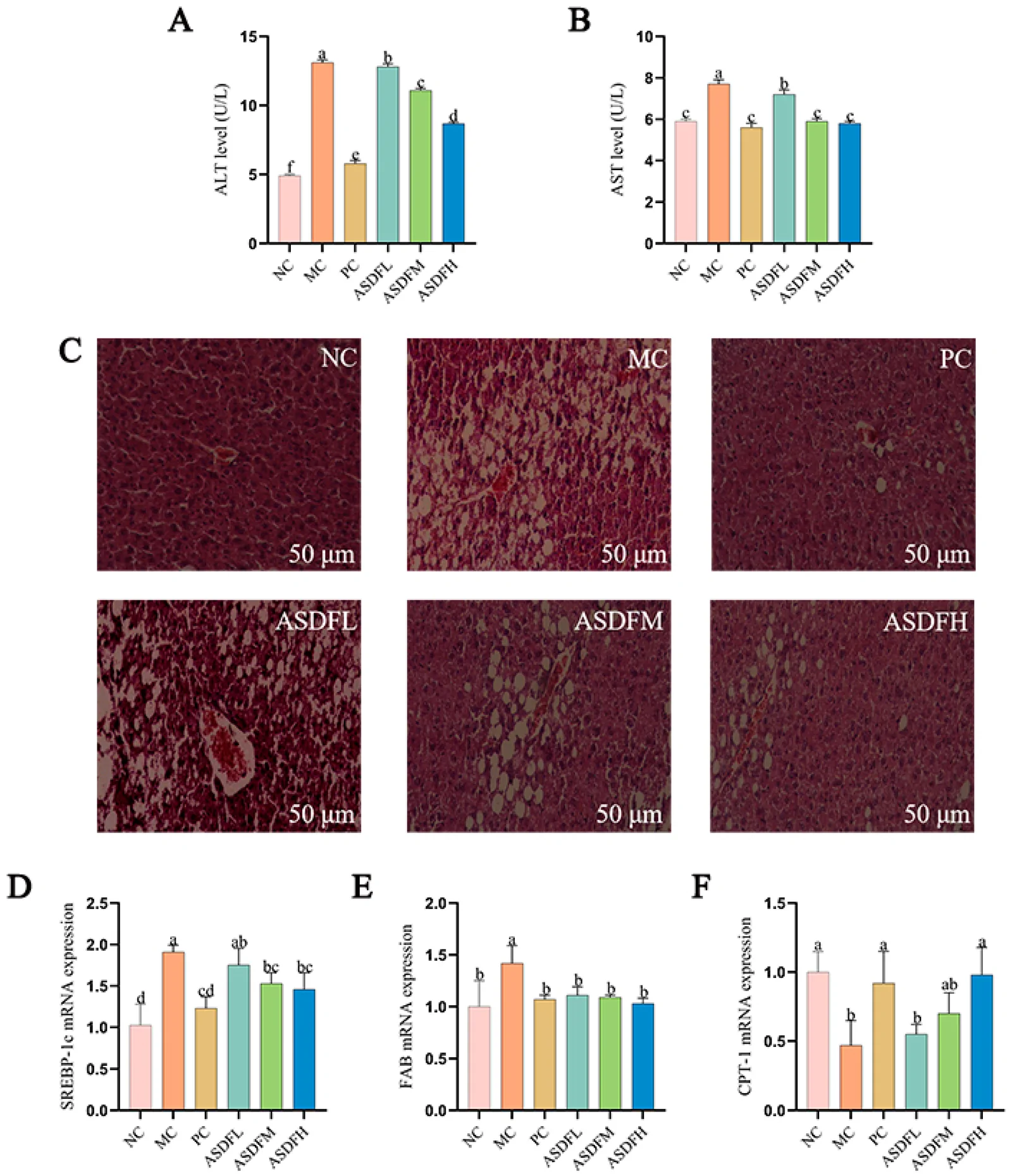
Effect of ASDF on liver function, pathology, and lipid metabolism-related gene expression in HFD-fed mice; (**A**) Serum ALT level, (**B**) Serum AST level, (**C**) Representative images of liver histopathology stained with H&E (scale bar: 50 μm), (**D**) Relative mRNA expression of *SREBP-1c,* (**E**) Relative mRNA expression of *FAS*, (**F**) Relative mRNA expression of *CPT-1*. Means with different letters indicate significant differences (*p* < 0.05).

Excessive intake of dietary fat leads to abnormal accumulation of hepatic lipids, causing hepatic steatosis and subsequent liver injury [85]. ALT and AST activities are standard biomarkers for detecting early liver injury [86]. Compared with the NC group, the MC group exhibited severe liver dysfunction, with AST levels elevated by approximately 1.3-fold and ALT levels elevated by approximately 2.67-fold (Figure 6A,B). Compared with the MC group, the ASDF intervention groups showed significant reductions. For AST, the ASDFM and ASDFH groups reduced the levels to a degree with no significant difference from the NC and PC groups. For ALT, ASDF exhibited a clear dose-dependent reduction, among which the inhibitory effect of ASDFH was significantly stronger than that of ASDFM and ASDFL. Furthermore, histological observation via H&E staining (Figure 6C) showed that hepatocytes in the NC group were tightly arranged with normal morphology. In contrast, the MC group exhibited disrupted cellular structures and massive fat vacuoles, confirming severe hepatic steatosis. ASDF intervention dose-dependently alleviated these pathological changes, and the hepatocyte morphology and arrangement in the ASDFH group were highly similar to those of the NC group.

To further elucidate the underlying mechanism, the mRNA expression levels of key genes regulating hepatic lipid metabolism, including *SREBP-1c*, *FAS*, and *CPT-1*, were determined. *SREBP-1c* is a crucial nuclear transcription factor that binds to sterol regulatory elements in the promoters of lipogenic genes to activate transcription, thereby specifically regulating cholesterol and fatty acid metabolism [87]. *FAS* is a key downstream target of *SREBP-1c* and a potential therapeutic target for obesity [88]. *CPT-1* is a lipid catabolism-related gene and a key regulatory enzyme for long-chain fatty acid β-oxidation in the liver [89]. As shown in Figure 6D–F, compared with the NC group, the high-fat diet induced a severe lipogenic state in the MC group, with the mRNA levels of *SREBP-1c* and *FAS* upregulated by approximately 1.9-fold and 1.4-fold, respectively, while the lipid oxidation gene *CPT-1* was suppressed to about 45% of the NC level. Overall, ASDF intervention dose-dependently reversed these molecular abnormalities. Notably, the ASDFH group exhibited the most comprehensive regulatory effect, restoring *FAS* and *CPT-1* levels to the NC baseline. These results were consistent with recent findings that *Ganoderma lucidum* and *Abelmoschus esculentus* polysaccharides can ameliorate hepatic steatosis by regulating the *SREBP-1c*/*FAS* pathway and promoting *CPT-1*-mediated oxidation [90,91]. Thus, ASDF can effectively inhibit lipogenesis and enhance lipid oxidation, thereby alleviating high-fat diet-induced liver injury.

### 3.9. Effect of ASDF on Intestinal Flora in HFD-Fed Mice

Intestinal flora dysbiosis is closely associated with the pathogenesis of obesity and metabolic disorders [92]. Since the ASDFH group exhibited the strongest lipid-lowering effect in our dose–response experiments, ASDFH was selected for 16S rRNA sequencing alongside the NC and MC groups to explore the effects of ASDF on the intestinal flora of HFD-fed mice.

**Figure 7:**
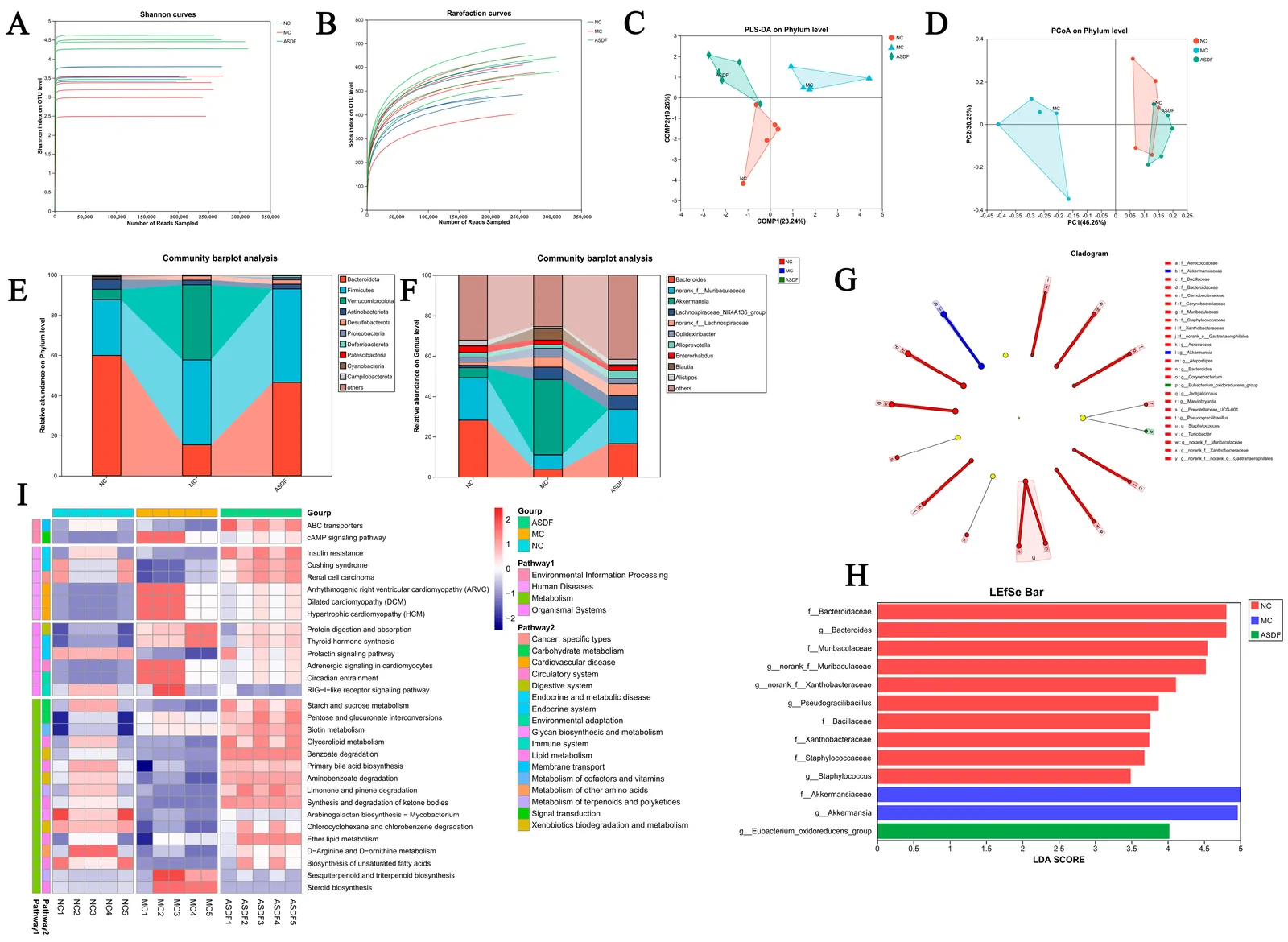
Effect of ASDF on the intestinal flora in HFD-fed mice; (**A**) Shannon diagram, (**B**) Sobs diagram, (**C**) PCoA diagram, (**D**) PLS-DA diagram, (**E**) Relative abundance of intestinal flora at the phylum level, (**F**) Relative abundance of intestinal flora at the genus level, (**G**) LEfSe statistical difference analysis cladogram, (**H**) LDA diagram, (**I**) The heatmap of functional prediction of altered intestinal flora by PICRUSt analysis based on KEGG.

As shown in Figure 7A,B, the Shannon and Sobs index curves plateaued, indicating that the sequencing depth was sufficient. Alpha diversity reflects the species richness and evenness within a single sample [93]. Specifically, the Shannon and Simpson indices evaluate community diversity, while the Ace and Chao indices estimate species richness. Compared with the NC group, the MC group exhibited significant decreases in Shannon, Ace, and Chao indices and a significant increase in the Simpson index (Table 3). Conversely, ASDF intervention significantly reversed these trends.

Beta diversity assesses the differences in species composition among different samples [94]. PLS-DA and PCoA were employed to visualize these structural variations. As shown in Figure 7C,D, the NC and MC groups were clearly separated, indicating that HFD induced a severe structural shift in the intestinal flora. However, the ASDF group clustered closely with the NC group, demonstrating that ASDF intervention could partially restore HFD-induced structural dysbiosis.

At the phylum level (Figure 7E), the intestinal flora was mainly composed of *Firmicutes*, *Bacteroidota*, and *Verrucomicrobiota*. Compared with the NC group, the relative abundance of *Firmicutes* in the MC group was increased by approximately 1.68-fold, while *Bacteroidota* was drastically reduced to nearly one-ninth. Additionally, *Verrucomicrobiota* was highly enriched in the MC group. The increased *Firmicutes*/*Bacteroidota* (*F*/*B*) ratio is a recognized indicator of obesity propensity [95]. ASDF intervention reversed these trends, bringing the percentages of these phyla closer to the NC group.

At the genus level (Figure 7F), HFD feeding induced a significant depletion of *Bacteroides* and *norank_f__Muribaculaceae*, accompanied by a marked enrichment of *Akkermansia* and *Blautia*, all of which were reversed by ASDF intervention. LEfSe and LDA analyses (>4) further confirmed *Akkermansia* as a characteristic genus of the MC group, whereas *Bacteroides* and *norank_f__Muribaculaceae* were enriched in the NC and treatment groups. *Eubacterium* was identified as a characteristic genus of the ASDF group (Figure 7G,H).

Mechanistically, *Bacteroides* species harbor abundant polysaccharide utilization loci and CAZymes, enabling them to catabolize indigestible dietary polysaccharides and improve obesity [96]. *Norank_f__Muribaculaceae* plays a major metabolic role in the anaerobic conversion of polysaccharides into short-chain fatty acids [97]. Regarding *Akkermansia*, although generally considered a probiotic candidate [98], its physiological impact is highly context- and abundance-dependent. Under HFD conditions, the proliferation of *Akkermansia* can be favored [99]. However, as a mucin-degrading bacterium residing in the mucus layer, an excessive abundance of *Akkermansia* can lead to over-degradation of intestinal mucin, potentially triggering an exaggerated immune response and causing intestinal barrier damage [100]. Therefore, the suppression of its overgrowth by ASDF may represent a restoration of gut ecological balance and barrier homeostasis. Furthermore, *Blautia* has been reported to be increased in HFD-fed mice [101], and is positively correlated with hepatic and serum TG levels [102]. Conversely, *Eubacterium*, enriched in the ASDF group, is a major butyrate producer that can convert cholesterol into fecal sterols to lower blood cholesterol [12,103]. These results indicate that ASDF intervention effectively alleviates HFD-induced intestinal flora dysbiosis.

Furthermore, PICRUSt software was used to predict the functional composition of the microbiota (Figure 7I). It must be emphasized that PICRUSt is a computational inference tool based on 16S rRNA data and cannot substitute for actual metagenomic sequencing. Therefore, these predicted pathways require further validation. The prediction revealed that pathways related to ABC transporters, insulin resistance, and glyceride metabolism were abnormally activated in the MC group compared to the NC group, which is consistent with the pathological features of obesity-related metabolic disorders [104].

In conclusion, ASDF can modulate the intestinal flora structure, significantly enriching *Bacteroides*, *norank_f__Muribaculaceae*, and *Eubacterium*, while simultaneously inhibiting the excessive proliferation of *Akkermansia* and *Blautia*, thus potentially regulating metabolic functions.

### 3.10. Effects of ASDF on Metabolism in HFD-Fed Mice

Serum circulating metabolites, which reflect host-microbiota co-metabolism, can participate in host physiological regulation through signaling molecules or signal transduction pathways, playing an important role in the pathogenesis of obesity and metabolic disorders [105]. To deeply investigate the intervention mechanism of ASDF on HFD-induced metabolic disorders, untargeted serum metabolomics was performed using LC-MS/MS.

**Figure 8:**
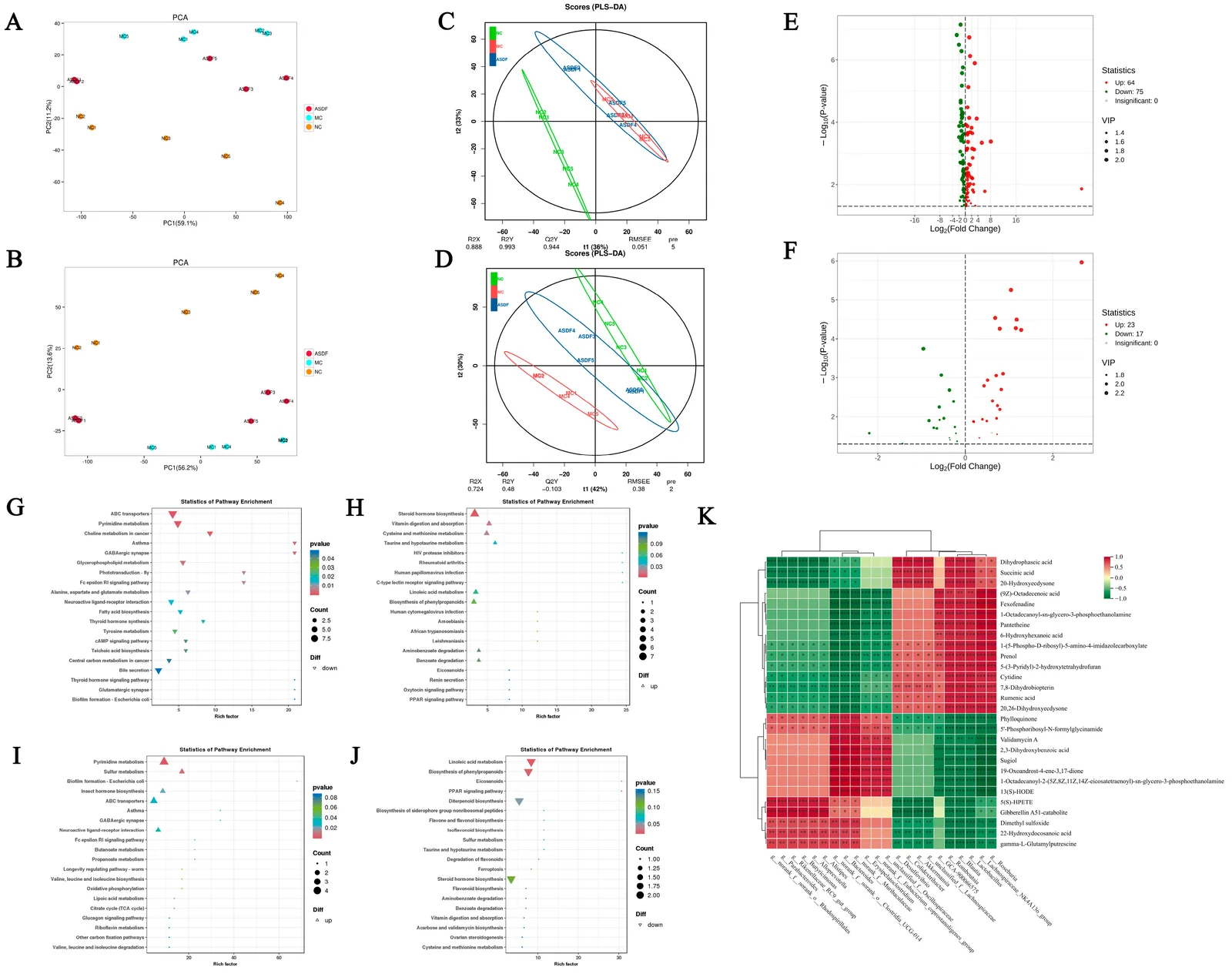
Regulation of metabolic disorders by ASDF in HFD-fed mice; (**A**) PCA score plots in the positive ion model, (**B**) PCA score plots in the negative ion model, (**C**) OPLS-DA scores plot in the positive ion model, (**D**) OPLS-DA scores plot in the negative ion model, (**E**) Volcano plots for differential metabolites in MC vs. NC, (**F**) Volcano plots for differential metabolites in ASDF vs. MC, (**G**) Up-regulated KEGG pathway in MC vs. NC, (**H**) Down-regulated KEGG pathway in MC vs. NC, (**I**) Down-regulated KEGG pathway in ASDF vs. MC, (**J**) Up-regulated KEGG pathway in ASDF vs. MC, (**K**) Spearman’s correlation analysis of the intestinal flora and metabolites, (* 0.01 < *p* ≤ 0.05, ** 0.001 < *p* ≤ 0.01, *** *p* ≤ 0.001).

Data quality assessment showed that the samples in the PCA in positive and negative ion modes showed good aggregation within the group (Figure 8A,B), indicating that the experimental process had ideal stability and reproducibility. PLS-DA showed a significant trend of intergroup separation in both positive and negative ion modes (Figure 8C,D), suggesting characteristic metabolic differences among the three groups. The above multidimensional statistical analysis results verified the reliability of the experimental data.

The potential biomarkers were screened based on the PLS-DA model, and VIP > 1, |FC| > 1 and *p* < 0.05 were set as the screening threshold. A total of 139 differential metabolites were identified between the NC and MC groups (Figure 8E), indicating severe metabolic disorders in HFD-fed mice. Notably, ASDF intervention effectively regulated these disorders (Figure 8F). Specifically, compared with the MC group, ASDF intervention significantly reversed the abnormal levels of 40 serum metabolites, comprising the downregulation of 23 metabolites, including Succinic acid, 20-Hydroxyecdysone and Taurine, and the upregulation of 17 metabolites, including 13(S)-HODE and Apigenin. The detailed information for all 40 of these differential serum metabolites is provided in Table 4.

Based on the pathway enrichment analysis (Figure 8G,H), metabolic pathways were severely disturbed under HFD intervention. Pathways such as ABC transporters and tyrosine metabolism were significantly upregulated [106,107]. The Rich factor of the bile secretion pathway reached an extreme value of 20.0, suggesting its central regulatory role in the adaptive response to dietary fat overload [108]. The combined analysis of Figure 8I,J revealed a systemic metabolic remodeling by ASDF. ASDF intervention reversed the HFD-induced abnormal activation of ABC transporters and bile secretion pathways, thereby restoring normal lipid transport and cholesterol efflux [109]. Furthermore, triglyceride metabolism and unsaturated fatty acid synthesis pathways were significantly activated in the MC group. ASDF reversed these pathways, suggesting an inhibition of de novo lipogenesis [110]. In terms of glucose metabolism, ASDF significantly improved insulin sensitivity by enhancing the cAMP-PKA signaling pathway [111]. The reduced activity of the primary bile acid synthesis pathway and the enhanced intestinal farnesoid X receptor (FXR) signaling in the ASDF group might explain the reduced serum total bile acid level [112]. Additionally, the HFD-induced inhibition of oxidative phosphorylation and TCA cycle pathways was significantly reversed by ASDF, restoring mitochondrial function and promoting energy expenditure [113,114,115,116]. Notably, the predicted gene function of intestinal flora under ASDF intervention was highly overlapping with metabolic pathways. Therefore, the intestinal flora and metabolites that were significantly regulated by ASDF were correlated (Figure 8K). ASDF intervention elevated beneficial genera and formed a functional synergistic network with their metabolites. Specifically, the enrichment of *Bacteroides* by ASDF was strongly negatively correlated with the level of serum Succinic acid [r = −0.79, *p* < 0.01], which aligns with the restoration of the TCA cycle. *Bacteroides* likely facilitates the consumption of succinate, preventing its accumulation and thereby improving mitochondrial energy metabolism [117].

Meanwhile, ASDF intervention significantly inhibited the metabolic activities associated with the excessive proliferation of specific genera under HFD conditions. The negative correlation between *Akkermansia* and serum 13(S)-HODE [r = −0.51, *p* < 0.05] indicated that the suppression of excessive *Akkermansia* by ASDF restored the concentration of 13(S)-HODE (an endogenous PPARγ ligand), thereby promoting fatty acid oxidation and inhibiting adipocyte differentiation [118]. Additionally, the overproliferation of *Akkermansia* was positively correlated with gamma-L-Glutamylputrescine, a metabolite associated with disrupted intestinal permeability [119]. Moreover, *Blautia* was positively correlated with serum 20-Hydroxyecdysone [r = 0.95, *p* < 0.01]. 20-Hydroxyecdysone is a natural antagonist of FXR. Its downregulation by ASDF (accompanied by decreased *Blautia*) may alleviate the FXR-mediated negative feedback inhibition of bile acids, thereby improving intestinal inflammation and lipid metabolism [120,121]. FXR is a core nuclear receptor for bile acid metabolism [122], and changes in *Blautia* abundance are closely related to FXR activity [123]. In conclusion, ASDF intervention significantly reshaped the serum metabolic profile in HFD-fed mice, primarily characterized by the downregulation of succinic acid and 20-hydroxyecdysone, and the upregulation of 13(S)-HODE. These key metabolic shifts, which are closely linked to specific intestinal flora changes, may be associated with the restoration of the TCA cycle, the activation of PPARγ-mediated lipid metabolism, and the regulation of FXR-mediated bile acid homeostasis.

## 4. Conclusions

In this study, a structurally novel soluble dietary fiber was extracted from *A. heimuer* residue. ASDF exhibited a semi-crystalline nature and a unique porous hollow spherical morphology, which endowed it with excellent thermal stability and strong in vitro capacities for binding bile salts and inhibiting pancreatic lipase. Meanwhile, ASDF effectively ameliorated HFD-induced obesity, hepatic steatosis, and dyslipidemia. Furthermore, ASDF modulated the gut microbiota structure by enriching *Bacteroides* and *norank_f__Muribaculaceae*, while suppressing the excessive proliferation of *Akkermansia* and *Blautia*. Additionally, serum metabolomics revealed that ASDF significantly downregulated succinic acid and 20-hydroxyecdysone, while upregulating 13(S)-HODE.

This study provides a green strategy for the high-value utilization of agricultural waste and highlights the potential of ASDF as an effective functional dietary supplement for controlling obesity and metabolic syndrome.

However, some limitations remain. The precise structure of ASDF has not been fully elucidated. Future studies should employ methylation analysis and nuclear magnetic resonance spectroscopy to fully elucidate its exact glycosidic linkages. Second, in vitro cell models, such as Caco-2 and HepG2 cells, should be further employed to validate specific metabolic pathways and cellular mechanisms. Finally, future studies should utilize mono-colonization experiments, FMT, and direct gavage of specific metabolites to confirm in vivo causal relationships, thereby further elucidating its mechanism of action.

## Figures and Tables

**Table 1:** Primer sequences.

Primer Name	Forward Primer (5′→3′)	Reverse Primer (5′→3′)	Product Size (bp)
FAS	TGTCCTGCCTCTGGTGCTTG	GCAAAATGGGCCTCCTTGATAT	148
SREBP-1c	GAAACCCGAAGTGGTGGAGAC	TGGGCTTTGACCTGGCTATC	159
CPT-1	GCACGAGGAAAAAATAAGCAATCT	TGTCAAACCACCTGTCGAAAC	167

**Table 2:** Effects of ASDF on organ and adiposity indices in HFD-fed mice.

Sample	Liver Index	Heart Index	Spleen Index	Kidney Index	Inguinal Fat Index	Epididymal Fat Index
NC	2.672 ± 0.264 e	0.33 ± 0.081 de	0.157 ± 0.019 e	0.98 ± 0.146 b	1.384 ± 0.520 e	4.159 ± 0.932 d
MC	3.227 ± 0.225 a	0.470 ± 0.069 a	0.221 ± 0.047 a	1.068 ± 0.143 a	2.468 ± 0.687 a	6.083 ± 1.569 a
PC	2.856 ± 0.403 d	0.325 ± 0.062 e	0.147 ± 0.072 f	0.852 ± 0.017 c	1.294 ± 0.326 f	4.363 ± 1.138 d
ASDFL	3.192 ± 0.308 ab	0.391 ± 0.047 b	0.202 ± 0.035 b	1.00 ± 0.092 a	2.119 ± 0.371 b	6.153 ± 1.095 a
ASDFM	3.158 ± 0.488 b	0.376 ± 0.067 c	0.186 ± 0.169 d	0.99 ± 0.293 a	2.03 ± 0.567 c	5.712 ± 1.272 b
ASDFH	3.048 ± 0.476 c	0.345 ± 0.046 d	0.194 ± 0.048 c	0.978 ± 0.081 b	1.883 ± 0.281 d	5.329 ± 1.165 c

Note: Means with different letters indicate significant differences (*p* < 0.05).

**Table 3:** Alpha diversity analysis of HFD-fed mice.

Sample	Shannon	Simpson	Ace	Chao
NC	3.455 ± 0.125 a	0.031 ± 0.009 c	542.364 ± 11.323 a	543.851 ± 16.337 a
MC	2.921 ± 0.203 c	0.084 ± 0.005 a	496.519 ± 17.132 c	510.797 ± 28.321 c
ASDF	3.108 ± 0.227 b	0.058 ± 0.006 b	526.999 ± 21.468 b	521.901 ± 21.342 b

Note: Means with different letters indicate significant differences (*p* < 0.05).

**Table 4:** Serum differential metabolites reversed by ASDF intervention in HFD-fed mice.

Metabolites	Regulated:	log2FC	*p* Value	VIP	KEGG ID
3-Sulfino-L-alanine	down	(0.35)	0.04	1.67	C00606
Dimethyl sulfoxide	down	(0.59)	0.01	1.77	C11143
2,3-Dihydroxybenzoic acid	down	(0.27)	0.00	1.94	C00196
5′-Phosphoribosyl-N-formylglycinamide	down	(0.60)	0.01	2.06	C04376
19-Oxoandrost-4-ene-3,17-dione	down	(0.55)	0.00	2.15	C05297
5(S)-HPETE	down	(0.72)	0.02	1.91	C05356
20alpha-Hydroxy-4-pregnen-3-one	down	(0.24)	0.02	1.80	C04042
1-Octadecanoyl-2-(5Z,8Z,11Z,14Z-eicosatetraenoyl)-sn-glycero-3-phosphoethanolamine	down	(0.21)	0.03	1.70	C21480
13(S)-HODE	down	(0.36)	0.00	2.13	C14762
12,13-DHOME	down	(0.19)	0.04	1.72	C14829
Gibberellin A51-catabolite	down	(0.96)	0.00	2.33	C11854
Validamycin A	down	(0.36)	0.03	1.68	C12112
Apigenin	down	(1.43)	0.05	1.71	C01477
22-Hydroxydocosanoic acid	down	(0.65)	0.02	1.91	C19623
Sugiol	down	(0.49)	0.01	1.94	C21822
gamma-L-Glutamylputrescine	down	(2.19)	0.03	1.84	C15699
Phylloquinone	down	(0.83)	0.01	2.02	C02059
P1,P4-Bis(5′-uridyl) tetraphosphate	up	0.72	0.03	1.69	C06198
Xanthurenic acid	up	0.79	0.01	1.99	C02470
Succinic acid	up	0.86	0.00	2.18	C00042
Taurine	up	0.19	0.01	1.87	C00245
1-(5-Phospho-D-ribosyl)-5-amino-4-imidazolecarboxylate	up	0.19	0.01	1.89	C04751
dTDP	up	0.71	0.01	1.89	C00363
4-Methyl-2-oxopentanoate	up	0.49	0.01	1.85	C00233
6-Hydroxyhexanoic acid	up	0.39	0.01	1.88	C06103
Uridine	up	0.51	0.00	2.06	C00299
Fexofenadine	up	1.04	0.00	2.35	C06999
1-Octadecanoyl-sn-glycero-3-phosphoethanolamine	up	0.79	0.00	2.30	C21484
Dihydrophaseic acid	up	1.28	0.00	2.31	C15971
Cytidine	up	0.43	0.00	2.08	C00475
(9Z)-Octadecenoic acid	up	0.32	0.03	1.69	C00712
7,8-Dihydrobiopterin	up	0.68	0.00	2.30	C02953
5-(3-Pyridyl)-2-hydroxytetrahydrofuran	up	1.15	0.00	2.32	C19578
Leukotriene D4	up	2.66	0.00	2.35	C05951
Guanosine 3′,5′-bis(diphosphate)	up	0.61	0.03	1.66	C01228
Pantetheine	up	0.62	0.00	2.01	C00831
Prenol	up	0.82	0.00	2.08	C01390
20-Hydroxyecdysone	up	1.17	0.00	2.32	C02633
20,26-Dihydroxyecdysone	up	0.74	0.01	1.95	C16500
Rumenic acid	up	0.71	0.00	2.14	C04056

Note: The listed metabolites were significantly altered in the model group (MC vs. NC) and subsequently exhibited opposite regulatory trends following ASDF treatment (ASDF vs. MC). The “Up” and “Down” directions indicate the relative changes in metabolite levels in the ASDF group compared to the MC group (“Up” means the level was higher in ASDF than in MC, representing a reversal of an HFD-induced decrease; “Down” means the level was lower in ASDF than in MC, representing a reversal of an HFD-induced increase). The numbers in parentheses were negative values. Differential metabolites were screened based on VIP > 1, |FC| > 1, and *p* < 0.05.

## Data Availability

The original contributions presented in this study are included in the article. Further inquiries can be directed to the corresponding authors.

## Abbreviations

HLP: Hyperlipidemia; HFD: High-fat diet; ASDF: *Auricularia heimuer* soluble dietary fiber; ASDFL: ASDF low-dose group; ASDFM: ASDF medium-dose group; ASDFH: ASDF high-dose group; NC: Normal control; MC: Model control; PC: Positive control; ALT: Alanine aminotransferase; AST: Aspartate aminotransferase; TC: Total cholesterol; TG: Triglyceride; HDL-C: High-density lipoprotein cholesterol; LDL-C: Low-density lipoprotein cholesterol; 4-MUO: 4-Methylumbelliferyl oleate; IC_50_: Half-maximal inhibitory concentration; FT-IR: Fourier transform infrared spectroscopy; XRD: X-ray diffraction; TGA: Thermogravimetric analysis; DTG: Derivative thermogravimetry; SEM: Scanning electron microscopy; AFM: Atomic force microscopy; RT-qPCR: Reverse transcription quantitative polymerase chain reaction; PCA: Principal component analysis; PCoA: Principal coordinate analysis; OPLS-DA: Orthogonal partial least squares-discriminant analysis.
